# Effects of the multidomain intervention with nutritional supplements on cognition and gut microbiome in early symptomatic Alzheimer’s disease: a randomized controlled trial

**DOI:** 10.3389/fnagi.2023.1266955

**Published:** 2023-11-02

**Authors:** Eun Hye Lee, Geon Ha Kim, Hee Kyung Park, Hae Jin Kang, Yoo Kyoung Park, Hye Ah Lee, Chang Hyung Hong, So Young Moon, Woorim Kang, Hyun-Seok Oh, Hai-Jeon Yoon, Seong Hye Choi, Jee Hyang Jeong

**Affiliations:** ^1^Department of Neurology, Ewha Womans University School of Medicine, Seoul, Republic of Korea; ^2^Department of Neurology, Ewha Womans University Seoul Hospital, Seoul, Republic of Korea; ^3^Department of Neurology, Ewha Womans University Mokdong Hospital, Seoul, Republic of Korea; ^4^Division of Psychiatry, Department of Mental Health Care of Older People, University College London, London, United Kingdom; ^5^Department of Medical Nutrition (AgeTech-Service Convergence Major), Kyung Hee University, Yongin, Republic of Korea; ^6^Clinical Trial Center, Ewha Womans University Mokdong Hospital, Seoul, Republic of Korea; ^7^Department of Psychiatry, Ajou University School of Medicine, Suwon, Republic of Korea; ^8^Department of Neurology, Ajou University School of Medicine, Suwon, Republic of Korea; ^9^CJ Bioscience Inc., Seoul, Republic of Korea; ^10^Department of Nuclear Medicine, Ewha Womans University School of Medicine, Seoul, Republic of Korea; ^11^Department of Neurology, Inha University College of Medicine, Incheon, Republic of Korea

**Keywords:** Alzheimer’s disease, dementia, mild cognitive impairment, prevention, multidomain intervention, nutritional supplements, gut microbiome

## Abstract

**Background:**

The SoUth Korean study to PrEvent cognitive impaiRment and protect BRAIN health through lifestyle intervention in at-risk elderly people (SUPERBRAIN) is a part of the World-Wide Finnish Geriatric Intervention Study to Prevent Cognitive Impairment and Disability (WW-FINGERS) network. This study aimed to demonstrate the effects of the SUPERBRAIN-based multidomain intervention with nutritional supplements in amyloid positive emission tomography (PET) proven early symptomatic Alzheimer’s disease patients.

**Methods:**

Forty-six participants who were diagnosed with mild cognitive impairment or mild dementia and were positive in the amyloid PET study randomized into three groups: group A, the multidomain intervention with nutritional supplements; group B, nutritional supplements only; and a control group. The primary outcome was a change in the Repeatable Battery for the Assessment of Neuropsychological Status (RBANS) total scale index score after an 8-week intervention. Secondary outcomes, including gut microbiome data, were also analyzed.

**Results:**

The RBANS total scale index score improved significantly in group A compared with group B (*p* < 0.032) and compared with the control group (*p* < 0.001). After intervention, beta diversity of the gut microbiome between group A and the control group increased, and patients in group A were more enriched with *Bifidobacterium*.

**Conclusion:**

SUPERBRAIN-based multidomain intervention with nutritional supplements improves cognition and gut microbiota in patients with early symptomatic Alzheimer’s disease who were amyloid-positive by PET.

## 1. Introduction

Alzheimer’s disease (AD) and related dementias are the most common neurodegenerative diseases (World Health Organization, 2012, 2017). However, as curative pharmacological treatments for AD dementia are still lacking (Hung and Fu, 2017; Alzheimer’s Association, 2022), nonpharmacological treatments to prevent and modify cognitive impairment have been employed. Although single-domain interventions have shown modest outcomes and inconsistent results (Hill et al., 2017; de Souto et al., 2018; Radd-Vagenas et al., 2018; Ding et al., 2020), multidomain interventions have emerged as an alternative approach that considers the multifactorial causes of AD dementia (Coley et al., 2008; Scarmeas et al., 2009; Andrieu et al., 2015).

The Finnish Geriatric Intervention Study to Prevent Cognitive Impairment and Disability (FINGER) trial reported that participants who received 24 months of multidomain intervention had better cognitive outcomes than control participants (Ngandu et al., 2015). To better adapt this intervention for use in other countries (World Health Organization, 2019), the World-Wide FINGERS (WW-FINGERS) network was launched. As a part of this worldwide network, the SoUth Korean study to PrEvent cognitive impaiRment and protect BRAIN health through lifestyle intervention in at-risk elderly people (SUPERBRAIN) was established as a multidomain intervention adjusted to the Korean context (Park et al., 2020). The feasibility study of the SUPERBRAIN presented significant improvement in cognition in intervention groups compared with a control group (Moon et al., 2021), and its magnetic resonance imaging results showed interval cortical thickening in facility-based multidomain intervention groups compared with the other groups (Moon et al., 2022).

In contrast, other clinical trials, such as the Multidomain Alzheimer Preventive Trial (MAPT) and Prevention of Dementia by Intensive Vascular Care (PreDIVA) trials, did not reveal significant effects from the interventions (van Charante et al., 2016; Andrieu et al., 2017). These inconsistent results require explanation, considering the positive results from the FINGER trial because of its well-selected target population (Solomon et al., 2021). The ancillary study of the MAPT revealed that a positive amyloid status could indicate a target population for multidomain intervention (Delrieu et al., 2019). Furthermore, a subgroup analysis of the original FINGER study reported that *APOE* ε4 carriers, who may have higher levels of brain amyloid pathology, showed a beneficial effect of the multidomain intervention on general cognition and memory within the group compared to non-carriers (Solomon et al., 2018). These findings called for further studies of the effects of multidomain interventions in amyloid-positive participants.

Recent trials have focused on a population at risk of dementia; that is, those not yet diagnosed with dementia. However, multidomain intervention might also be effective for patients with dementia because components of multidomain interventions are still available as treatment options for patients with dementia (Livingston et al., 2017, 2020). Consequently, this study targeted individuals with amyloid PET-proven early symptomatic AD, encompassing those with mild cognitive impairment (MCI) as well as individuals with dementia with Clinical Dementia Rating Scale Sum of Boxes (CDR-SB) score of less than 5.

We also enhanced the SUPERBRAIN multidomain intervention protocol for nutrition. Despite receiving nutritional guidance, patients with cognitive impairment find it challenging to eat balanced meals every day. To compensate for this, a nutritional supplement in the form of a multinutrient drink was added to the nutrition protocol of the SUPERBRAIN.

Therefore, this study investigated whether the effects of the multidomain intervention with nutritional supplements are superior to nutritional supplements alone or to no intervention (the control group) and whether the effects of nutritional supplements are superior to those of the control group.

## 2. Materials and methods

### 2.1. Study design

This study was a single-center, outcome assessor–blinded randomized controlled trial with a three-arm parallel design, and the intervention period was 8 weeks. The participants in group A received multidomain intervention with nutritional supplements, whereas those in group B received only nutritional supplements. A waitlist control group was also established in which participants were informed that the multidomain intervention program would be provided to them after the study. The baseline study was executed within 8 weeks before the start of the intervention, and the final study was completed within 4 weeks after the end of the last intervention. This study was registered with the Clinical Research Information Service (KCT0007253).

The study was conducted in accordance with the International Conference on Harmonization Good Clinical Practices Guidelines (Dixon, 1998). Written informed consent was obtained from all participants before enrollment. The Ewha Womans University, Seoul Hospital Institutional Review Board (IRB) approved this trial (SEUMC 2020-08-008-001).

### 2.2. Participants

The minimum number of participants required for adequate statistical power was calculated based on the effect size of changes in the Repeatable Battery for the Assessment of Neuropsychological Status (RBANS) total scale index score from a previous study (Calapai et al., 2017). The calculation was performed using G*Power version 3.1.9.7 (Heinrich Heine University, Düsseldorf, Germany). For 80% of the statistical power, at least 13 participants were required in each group. Considering a drop-out rate of approximately 20%, the final sample size was determined to be 16 participants per group.

Participants were recruited from individuals who visited the outpatient clinic of Ewha Womans University Seoul Hospital because of cognitive decline from March 2, 2021, to August 13, 2022. The inclusion criteria were: older adults aged 60–85 years; clinically diagnosed with MCI (Albert et al., 2011) or probable AD dementia (McKhann et al., 2011); Korean Mini-Mental State Examination Second Edition (K-MMSE-2) score of 17–27 (Han et al., 2008; Baek et al., 2016); Clinical Dementia Rating (CDR) (Morris, 1991) score of 0.5–1; CDR-SB (Choi et al., 2003) score of 0.5–5; having a caregiver with whom they could attend interviews and programs; and the subject agrees to participate in the study with written consent.

Participants were excluded if they had organic brain diseases or degenerative diseases known to be major causes of cognitive decline, including brain tumor, stroke, normal pressure hydrocephalus, Parkinson’s disease, Lewy body dementia, vascular dementia, and autoimmune encephalitis. In addition, individuals were excluded if they had infectious or metabolic diseases that may cause cognitive impairment, such as neurosyphilis, AIDS dementia, vitamin B12 deficiency, folate deficiency, and hypothyroidism. Other exclusion criteria were major psychiatric illness, epilepsy with intractable seizures, acute illness or acute infectious disease, probable encephalopathy caused by chronic liver or kidney disease, chronic pulmonary or cardiovascular disease under treatment, and any medical condition that would prevent cooperation with the interventions. Participants were also excluded if they had significant vision or hearing impairment, were illiterate, were unable to cooperate until the end of the study, were unable to safely complete the exercise program, had concurrent participation in another intervention trial, or refused to participate in the study.

Amyloid PET scans were conducted on the recruited participants. Participants who were determined as having amyloid pathology were enrolled in the study. Ultimately, the enrolled participants were amyloid PET-proven early symptomatic AD with a CDR-SB score below 5.

### 2.3. Amyloid PET

We used ^18^F-florbetaben manufactured by DuChemBio Co., Ltd. (Seoul, Korea) following the approval process of the Korean Ministry of Food and Drug Safety. Delayed-phase 3D list-mode dynamic PET images were acquired over a 20-min period 90–110 min after the bolus injection of 308.12 ± 10.93 MBq ^18^F-florbetaben. A spiral computed tomography scan of the brain was performed with the following parameters: 120 kV, 30 mA, and a slice thickness of 1.0 mm to correct for attenuation in the PET emission data. The participants’ heads were fixed with a head holder and a vacuum fixation cushion to reduce motion artifacts. The standard PET data were reconstructed into a 128 × 128 matrix (voxel size: 3.18 × 3.18 × 2.02 mm^3^) using the built-in 3D ordered subset expectation maximization algorithm (iteration: 4; subset: 12).

A visual reading by a nuclear medicine specialist was used to determine amyloid PET positivity.

### 2.4. Randomization

Participants were randomized to each group in a 1:1:1 ratio. The permuted block randomization method was applied using a macro in SAS software (SAS Institute Inc., Cary, NC, USA) with a block size of six. Only the independent statistical specialists knew the whole allocation sequence. Outcome assessors were not involved in the interventions, and participants were prohibited from discussing their assigned group when they met the outcome assessors.

### 2.5. Nutritional supplements

Memory Pack Plus (Daesang Life Science Corporation, Korea) was used as the nutritional supplement for this study. It is a multinutrient drink designed for brain health that contains eicosapentaenoic acid, docosahexaenoic acid, and phosphatidylserine (Supplementary material 1). It is aseptically packed as a 150 mL carton, and each carton contains 150 kcal.

### 2.6. Interventions

The protocol and contents of the multidomain intervention were based on the facility-based multidomain intervention of SUPERBRAIN (Park et al., 2020), which includes the following five components: the monitoring and managing of metabolic and vascular risk factors, cognitive training, physical exercise, nutritional guidance, and motivational enhancement. The number and frequency of each session were adjusted for a shorter study period than the previous SUPERBRAIN. Education on vascular risk factors was provided at the first visit with a booklet for the participants. The blood pressure, alcohol drinking, smoking, body weight, and abdominal circumference of each participant were monitored, and the results were discussed with each participant every 4 weeks. Each week, exercise and cognitive training sessions were done twice on the same visit day. Cognitive training sessions and nutritional guidance sessions were conducted with individual participants, whereas exercise sessions were conducted in a group of two participants. The participants attended nutritional guidance sessions once every 3 weeks. The details of the activities are described in Supplementary material 2. The motivation program was given as an in-person session at the beginning of the intervention and was followed by weekly text messages sent by the study coordinator.

Memory Pack Plus cartons were provided to group A and B participants. Both groups were told to drink two cartons daily. Participants in the control group received dementia-prevention education from a guideline booklet at the beginning of the study, and general medical care was provided to them.

### 2.7. Adherence and adverse events

Adherence to cognitive training, exercise, vascular-risk-factor monitoring, and nutritional guidance was assessed using the cumulative attendance rate for the 8-week intervention. The adherence to intake of nutritional supplements was assessed by calculating the number of remaining supplement cartons.

Adherence (%) to nutrient supplements $=\left(1-\frac{\mathrm{number}\;\mathrm{of}\;\mathrm{remaining}\;\mathrm{nutrient}\;\mathrm{supplements}}{\mathrm{number}\;\mathrm{of}\;\mathrm{distributed}\;\mathrm{nutrient}\;\mathrm{supplements}}\right)\times 100$ The study coordinator monitored the occurrence of adverse events.

### 2.8. Primary outcome

The primary outcome was a change in the total scale index score of the RBANS from baseline to after intervention and using a reference population of Korean adults to normalize the data (Randolph et al., 1998). We also evaluated five subdomain index scores: immediate memory, delayed memory, visuoconstruction, language and attention. Higher scores indicate better performance for all index scores.

### 2.9. Secondary outcomes

The secondary outcomes included global cognition, evaluated using K-MMSE-2 and CDR-SB. We also evaluated activities of daily living by the Korean Instrumental Activities of Daily Living scale (K-IADL) (Chin et al., 2018), depression by the 15-item Geriatric Depression Scale (GDS-15), and caregiver burden by the Zarit Burden Interview (ZBI). Physical performance was evaluated using the Short Physical Performance Battery (SPPB), grip power, and 30-s sit-to-stand test (endurance evaluation). Body composition was assessed using the body mass index and measurements of body fat, skeletal muscle mass, and visceral fat. The Nutrition Quotient for Elderly (NQ-E) was used to assess consumption of vegetables, fruits, beans, fish, milk, dairy products, eggs, water, fast food, pastries, and sweet food (Chung et al., 2018). Higher scores indicate better performance for K-MMSE-2, SPPB, and NQ-E. Lower scores indicate better performance for CDR-SB, K-IADL, GDS-15, and ZBI.

The secondary outcomes were modified from the first clinical trial enrollment. According to the results of the previous feasibility study of SUPERBRAIN (Moon et al., 2021), outcomes related to cognition, caregiver burden and physical performance were added. To assess nutrition, we replaced the mini-nutrition assessment that was a registered item in the initial trial registration with the NQ-E, which had shown a significant effect in the feasibility study. These changes were reported to the IRB, and further approval was obtained.

### 2.10. Exploratory outcomes

Fasting blood samples were collected from all participants in serum separation tubes and K2EDTA tubes. Total plasma cortisol and serum brain-derived neurotrophic factor (BDNF) were measured at baseline and after the interventions. BDNF was measured by Human Free BDNF enzyme-linked immunosorbent assay kit (R&D Systems, Minneapolis, MN, USA), and cortisol was measured using an ADVIA Centaur chemiluminescence immunoassay kit (Siemens Healthcare GmbH, Munich, Germany).

Stool collection kits containing buffer (CJ Bioscience Inc., Seoul, Korea) were provided to participants, and they were instructed on how to collect stools. Stool was collected 0–2 days before the baseline and final study visits. The kits were stored at room temperature and were shipped to CJ Bioscience Inc. for analysis within 1 week of the collection date.

We conducted 16S ribosomal RNA (rRNA) gene sequencing, taxonomic profiling, and functional profiling. The V3–V4 hypervariable region of the 16S rRNA gene was amplified with primers 341F and 805R using the direct polymerase chain reaction method. NEBNext Ultra II FS DNA Library Prep Kit for Illumina (New England Biolabs Inc., Ipswich, MA, USA) was used to construct DNA libraries. Sequencing of prepared DNA libraries by CJ Bioscience Inc. was conducted using the Illumina MiSeq platform (Illumina, San Diego, CA, USA) with 2 × 300 base pair kits.

The paired-end raw 16S rRNA sequence data were uploaded to the EzBioCloud and processed using a web-based EzBioCloud microbiome taxonomic profile tool.^1^ High-quality sequence reads were assigned to the “species group” at 97% sequence similarity using the PKSSU4.0 database.

Sex is a potential confounder of the microbiome outcome as it may influence both behavior that related to adherence to intervention components and the gut microbiome (Shobeiri et al., 2022). We confirmed that there were no significant differences in demographic characteristics, including sex, between the three experimental groups before conducting the microbiome analysis, thus controlling for the effect of sex.

Electroencephalogram data were obtained, but the data could not be read because of a technical problem. Therefore, analysis of electroencephalogram data was omitted from this study. This change was reported to the IRB, and further approval was obtained.

### 2.11. Statistical analyses

Statistical analyses were performed using a modified intention-to-treat approach. Baseline characteristics of each group were analyzed using analysis of variance (ANOVA) for continuous variables and chi-squared tests for categorical variables. To assess the effect of each intervention, outcomes were analyzed using a linear mixed-effect model. Comparisons between two groups were conducted using Bonferroni-adjusted *post hoc* analyses. Subgroup analyses for RBANS index scores were performed by disease stages (MCI and mild dementia) and sex. The analyses were performed using SPSS version 25.0 (IBM Corp., Armonk, NY, USA).

For gut microbiome analyses, the EzBioCloud Media Transfer Protocol server was used. The alpha diversity was calculated using the Chao1 and Shannon matrices, and its significance was assessed by Mann–Whitney U test. The generalized UniFrac metric was used for calculating the beta diversity, and the significance was assessed with permutational multivariate analysis of variance (PERMANOVA). Linear discriminant analysis effect size was performed to investigate the taxonomic differences among the groups. We also compared three experimental groups with an external healthy population (healthy control), and this microbiome analyses were performed using the Ez-Mx platform (CJ Bioscience Inc., Seoul, Korea) (Oh et al., 2022).

Statistical significance for each analysis was set at *p* < 0.05.

## 3. Results

### 3.1. Baseline characteristics of participants

**Figure 1:**
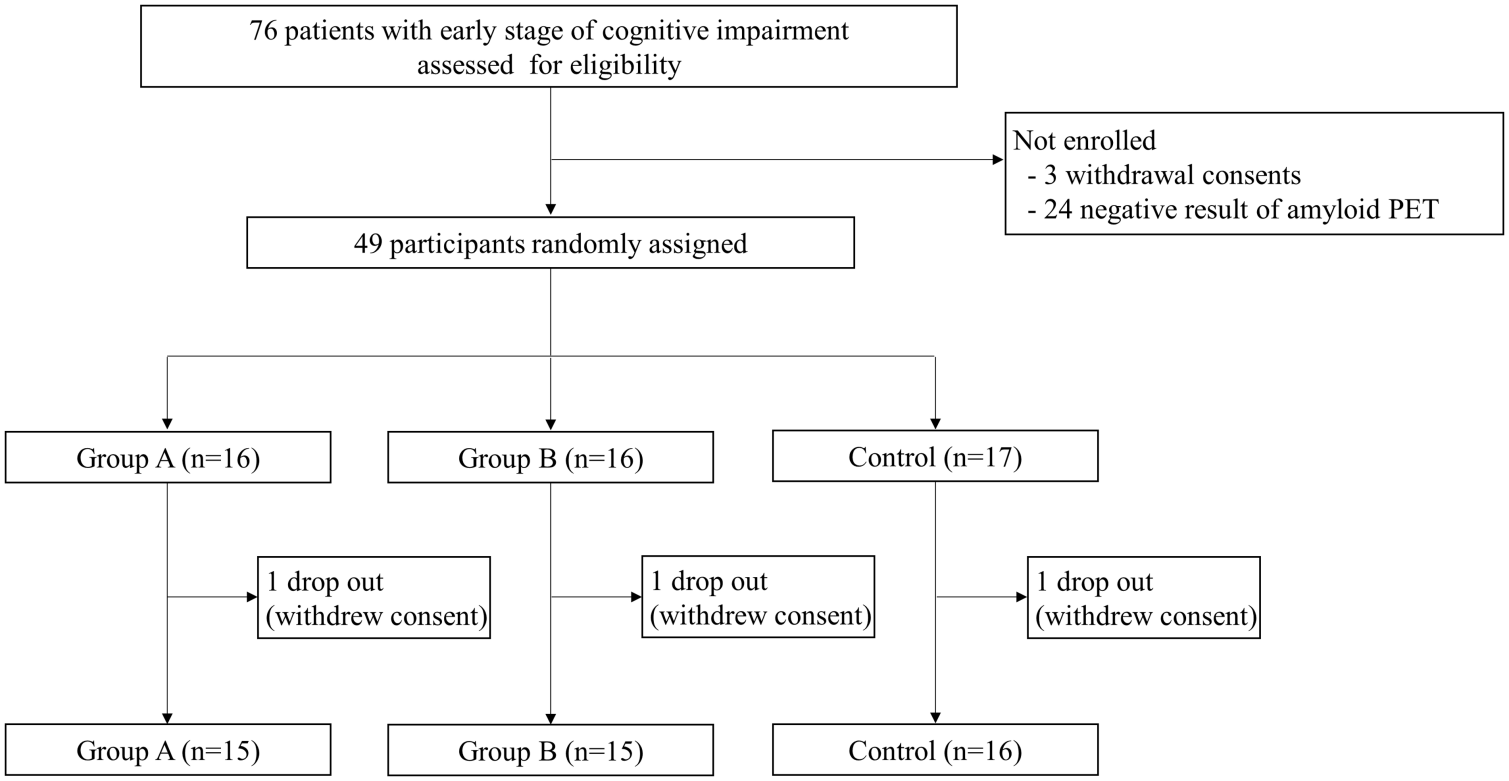
Study design. A total of 76 patients were screened for eligibility. Of those, 27 patients were not enrolled due to consent withdrawal or negative amyloid PET results. Thus, 49 participants with early-stage Alzheimer’s disease were randomly divided into three groups. Of these, three participants (one from each group) dropped out due to withdrawal of consent, and 46 participants completed the study.

Between October 12, 2020, and November 23, 2021, 76 patients were assessed for eligibility: 3 withdrew consent, and 24 failed screening due to negative amyloid PET results. We randomly assigned 49 participants with positive amyloid PET results and who were diagnosed with MCI or mild dementia to three groups: group A (*n* = 16), group B (*n* = 16), and the control group (*n* = 17). After randomization, three participants, one from each group, withdrew consent before the start of the interventions (Figure 1).

There were no significant differences in sex, age, education, and frequency of *APOE* ε4 carriers among the three groups. There were no significant differences among the three groups regarding the number of participants with diabetes mellitus, hyperlipidemia, or history of cardiac disease or stroke, but the number of participants with hypertension was significantly different among the three groups. The presence of hypertension was adjusted as a covariate in the analyses of primary and secondary outcomes. Cognitive function, physical performance, and nutritional status were similar among the three groups at baseline (Tables 1–3 and Supplementary material 3).

**Table 1:** Baseline characteristics of participants.

	Group A (*n* = 16)	Group B (*n* = 16)	Control (*n* = 17)	*p*
Sex, female	10 (62.5)	6 (37.5)	13 (76.5)	0.071
Age, years^†^	74.81 (5.74)	76.06 (5.64)	75.77 (6.67)	0.830
Education, year^†^	9.72 (3.56)	11.03 (4.53)	8.68 (4.57)	0.291
*APOE* ε4 carrier	8 (50.0)	6 (40.0)	4 (23.5)	0.283
HTN	7 (43.8)	4 (25.0)	12 (70.6)	0.031^*^
DM	5 (31.2)	5 (31.2)	8 (47.1)	0.550
Dyslipidemia	7 (43.8)	3 (18.8)	8 (47.1)	0.188
Cardiac disease	1 (6.2)	1 (6.2)	1 (5.9)	0.999
Stroke	0 (0.0)	0 (0.0)	1 (5.9)	0.383
Smoking	2 (12.5)	0 (0.0)	0 (0.0)	0.116
Alcohol consumption^‡^	0 (0.0)	1 (6.2)	0 (0.0)	0.349
CDR				0.946
0.5	12 (75.0)	12 (75.0)	12 (70.6)	
1	4 (25.0)	4 (25.0)	5 (29.4)	
AChEI use	14 (87.5)	15 (93.8)	17 (100.0)	0.326

Values are expressed as number (percent), except where noted otherwise.^*^p < 0.05.

† Values are expressed as mean (standard deviation).

‡ Drinking alcohol more than four times a day or for seven times a week.

AChEI, acetylcholinesterase inhibitor; CDR, Clinical Dementia Rating; DM, Diabetes mellitus; HTN, hypertension. We highlighted significant or borderline-significant values in bold in the letter.

**Table 2:** Mean changes in the scores of the RBANS.

	Baseline				Changes from baseline to study end							
	Group A (*n* = 15)	Group B (*n* = 15)	Control (*n* = 16)	*p*	Group A (*n* = 15)	Group B (*n* = 15)	Control (*n* = 16)	*p*	Post-hoc			
									A vs. B	A vs. C	B vs. C	
Total scale index score	78.60 (16.89)	74.67 (11.54)	79.50 (15.88)	0.332	9.00 (5.15, 12.85)	1.80 (−2.05, 5.65)	−4.56 (−8.29, −0.83)	<0.001^*^	0.032^*^	<0.001^*^	**0.063** ^**†**^	A > B A > C
Immediate memory	81.07 (12.81)	74.53 (11.11)	81.81 (14.41)	0.360	5.20 (−0.6, 11)	4.33 (−1.46, 10.13)	−0.56 (−6.18, 5.05)	0.306	>0.999	0.471	0.681	
Delayed memory	68.40 (19.99)	65.40 (18.45)	64.56 (17.41)	0.663	7.53 (1.44, 13.62)	3.73 (−2.36, 9.82)	0.06 (−5.83, 5.96)	0.218	>0.999	0.248	>0.999	
Visuo-construction	99.00 (14.81)	98.00 (12.71)	97.75 (15.70)	0.791	3.80 (−1.33, 8.93)	−3.40 (−8.53, 1.73)	−11.44 (−16.4, −6.47)	<0.001^*^	0.155	<0.001^*^	**0.085** ^**†**^	A > C
Language	85.87 (13.80)	87.33 (12.87)	93.81 (9.45)	0.055	6.20 (0.76, 11.64)	6.13 (0.7, 11.57)	−1.63 (−6.89, 3.64)	**0.066** ^**†**^	>0.999	0.129	0.134	
Attention	92.20 (12.81)	91.87 (19.41)	91.44 (15.17)	0.650	6.07 (1.54, 10.59)	−0.8 (−5.33, 3.73)	−1.13 (−5.51, 3.26)	0.046^*^	0.108	**0.079** ^**†**^	>0.999	

Values of baseline index scores are expressed as mean (standard deviation). Values of the changes in index scores are expressed as the adjusted mean (95% CI).Higher scores indicate better performance for all index scores.^*^p < 0.05.

† 0.05 < *p* < 0.1, borderline significance.

A vs. B, group A versus group B. We highlighted significant or borderline-significant values in bold in the letter; A vs. C, group A versus control group; B vs. C, group B versus control group; CI, confidence interval; RBANS, Repeatable Battery for the Assessment of Neuropsychological Status.

**Table 3:** Mean changes in the secondary outcomes.

	Baseline				Changes from baseline to study end							
	Group A (*n* = 15)	Group B (*n* = 15)	Control (*n* = 16)	*p*	Group A (*n* = 15)	Group B (*n* = 15)	Control (*n* = 16)	*p*	Post-hoc			
									A vs. B	A vs. C	B vs. C	
Cognitive function & Caregiver burden												
MMSE^†^	23.73 (3.17)	21.53 (2.50)	22.75 (3.38)	0.153	1.53 (0.42, 2.65)	0.67 (−0.45, 1.78)	−0.75 (−1.83, 0.33)	0.016^*^	0.818	0.014^*^	0.215	A > C
CDR-SB	2.93 (1.35)	3.53 (0.86)	3.44 (1.30)	0.373	0.03 (−0.09, 0.16)	0.00 (−0.13, 0.13)	0.16 (0.03, 0.28)	0.185	>0.999	0.513	0.251	
K-IADL	0.41 (0.30)	0.45 (0.18)	0.54 (0.31)	0.396	0.00 (−0.03, 0.03)	0.03 (0.00, 0.06)	0.03 (0.00, 0.05)	0.344	0.603	0.633	>0.999	
GDS-15	3.87 (4.00)	2.67 (2.74)	3.31 (3.03)	0.664	−0.13 (−1.34, 1.07)	0.33 (−0.87, 1.54)	−0.94 (−2.10, 0.23)	0.310	>0.999	>0.999	0.401	
ZBI	11.67 (10.89)	16.47 (12.11)	19.94 (15.91)	0.079	−0.8 (−4.88, 3.28)	−0.13 (−4.21, 3.95)	1.75 (−2.20, 5.70)	0.644	>0.999	>0.999	>0.999	
Physical performances												
Left grip (kg)^†^	19.20 (6.93)	21.76 (7.17)	18.38 (8.24)	0.435	1.37 (0.01, 2.74)	1.16 (−0.21, 2.52)	0.18 (−1.10, 1.45)	0.389	>0.999	0.611	0.888	
Right grip (kg)^†^	20.54 (6.88)	23.81 (7.02)	19.63 (7.66)	0.252	1.21 (−0.28, 2.69)	0.27 (−1.21, 1.75)	−0.36 (−1.75, 1.02)	0.305	>0.999	0.379	>0.999	
Sit-to-stand (times/30 s)^†^	11.67 (3.74)	12.33 (3.13)	13.06 (4.39)	0.597	1.98 (0.65, 3.30)	0.17 (−1.16, 1.50)	−0.44 (−1.68, 0.81)	0.031^*^	0.179	0.032^*^	>0.999	A > C
SPPB total score^†^	9.23 (0.42)	9.67 (0.43)	10.43 (0.41)	0.156	1.35 (0.63, 2.06)	−0.48 (−1.2, 0.24)	−0.94 (−1.61, −0.26)	<0.001^*^	0.002^*^	<0.001^*^	>0.999	A > B A > C
Body composition & Nutrition												
BMI (kg/m^2^)	22.96 (4.05)	23.21 (2.17)	23.75 (1.91)	0.729	0.15 (−0.25, 0.55)	0.16 (−0.25, 0.58)	0.06 (−0.33, 0.45)	0.920	>0.999	>0.999	>0.999	
Body fat (%)	33.63 (9.66)	29.30 (6.98)	34.19 (3.61)	0.176	−0.27 (−1.50, 0.96)	0.56 (−0.72, 1.83)	−0.06 (−1.25, 1.13)	0.626	>0.999	>0.999	>0.999	
SMM (kg)^†^	20.22 (3.74)	20.14 (4.93)	20.90 (4.47)	0.295	0.13 (−0.28, 0.54)	0.24 (−0.18, 0.66)	0.08 (−0.32, 0.47)	0.842	>0.999	>0.999	>0.999	
VF (level)	9.47 (4.47)	7.47 (2.26)	9.25 (2.70)	0.200	0.00 (−0.72, 0.72)	0.42 (−0.32, 1.16)	0.00 (−0.69, 0.69)	0.639	>0.999	>0.999	>0.999	
NQ-E^†^	56.52 (11.55)	61.98 (9.13)	60.85 (9.87)	0.312	6.17 (3.16, 9.18)	−0.25 (−3.26, 2.76)	−0.01 (−2.91, 2.90)	0.005^*^	0.012^*^	0.014^*^	>0.999	A > B A > C

Values of baseline index scores are expressed as mean (standard deviation). Values of the changes in index scores are expressed as the adjusted mean (95% CI).^*^p < 0.05.

† Higher scores indicate better performance for all index scores.

A vs. B, group A versus group B; A vs. C, group A versus control group; B vs. C, group B versus control group; CDR-SB, Clinical Dementia Rating Sum of Boxes; CI, confidence interval; GDS-15, Geriatric Depression Scale-15 items; K-IADL, Korean Instrumental Activities of Daily Living; MMSE, Korean Mini-Mental State Examination 2nd Edition; NQ-E, Nutrition Quotient for elderly; SPPB, Short Physical Performance Battery; BMI, Body Mass Index; SMM, Skeletal Muscle Mass; VF, Visceral Fat; ZBI, Zarit’s Burden Index. We highlighted significant or borderline-significant values in bold in the letter.

### 3.2. Adherence and adverse events

In group A, the adherence rates were 96.1% for the cognitive program, 94.0% for the exercise program, 100.0% for the vascular risk-factor monitoring, and 100.0% for the nutritional guidance. The adherence rate for the intake of nutritional supplements was 99.1% in group A and 83.7% in group B.

In group A, male participants showed adherence rates of 93.8% for the cognitive program, 87.5% for the exercise program, and 100.0% for supplement intake, while female participants demonstrated adherence rates of 99.5, 99.5, and 98.9%, respectively. In Group B, adherence rates for nutritional supplement intake were 83.3% for males and 84.8% for females.

No adverse event was reported during the entire intervention period.

### 3.3. Primary outcome

At baseline, there were no differences in the RBANS total scale index score and subdomain index scores among the three groups (Table 2). After intervention, the linear mixed-effect model revealed a significant difference in changes in RBANS total scale index score among the three groups. The adjusted means (95% confidence intervals) were as follows: group A, 9.00 (5.15, 12.85); group B, 1.80 (−2.05, 5.65); and control group, −4.56 (−8.29, 0.83); *p* < 0.001, effect size f^2^ = 0.530. *Post hoc* analysis revealed that the total scale index scores improved more for group A than for group B (*p* = 0.032) and more for group A than for the control group (*p* < 0.001). The visuoconstruction domain index score improved significantly different among the groups (*p* < 0.001), and *post hoc* analysis showed that the index score of group A improved more than that of the control group (*p* < 0.001, effect size *f*^2^ = 0.362). In the attention domain, the difference among the three groups was significant (*p* = 0.046), but a *post hoc* analysis found only a borderline significance in that group A improved more than the control group (*p* = 0.079) (Table 2).

### 3.4. Secondary outcomes

At baseline, there were no differences among the three groups for the K-MMSE-2, K-IADL, GDS-15, ZBI, physical performance, body composition, and nutrition score (Table 3).

After intervention, changes in K-MMSE-2 scores differed significantly among the three groups (*p* = 0.016) and the changes were the highest in treatment group A (*p* = 0.014) (Table 3). The change in the sit-to-stand time was significantly improved in group A compared with the control group (*p* = 0.032). The SPPB score improved significantly in group A compared with group B (*p* = 0.002) and in group A compared with the control group (*p* < 0.001) (Table 3).

### 3.5. Exploratory outcomes

**Figure 2:**
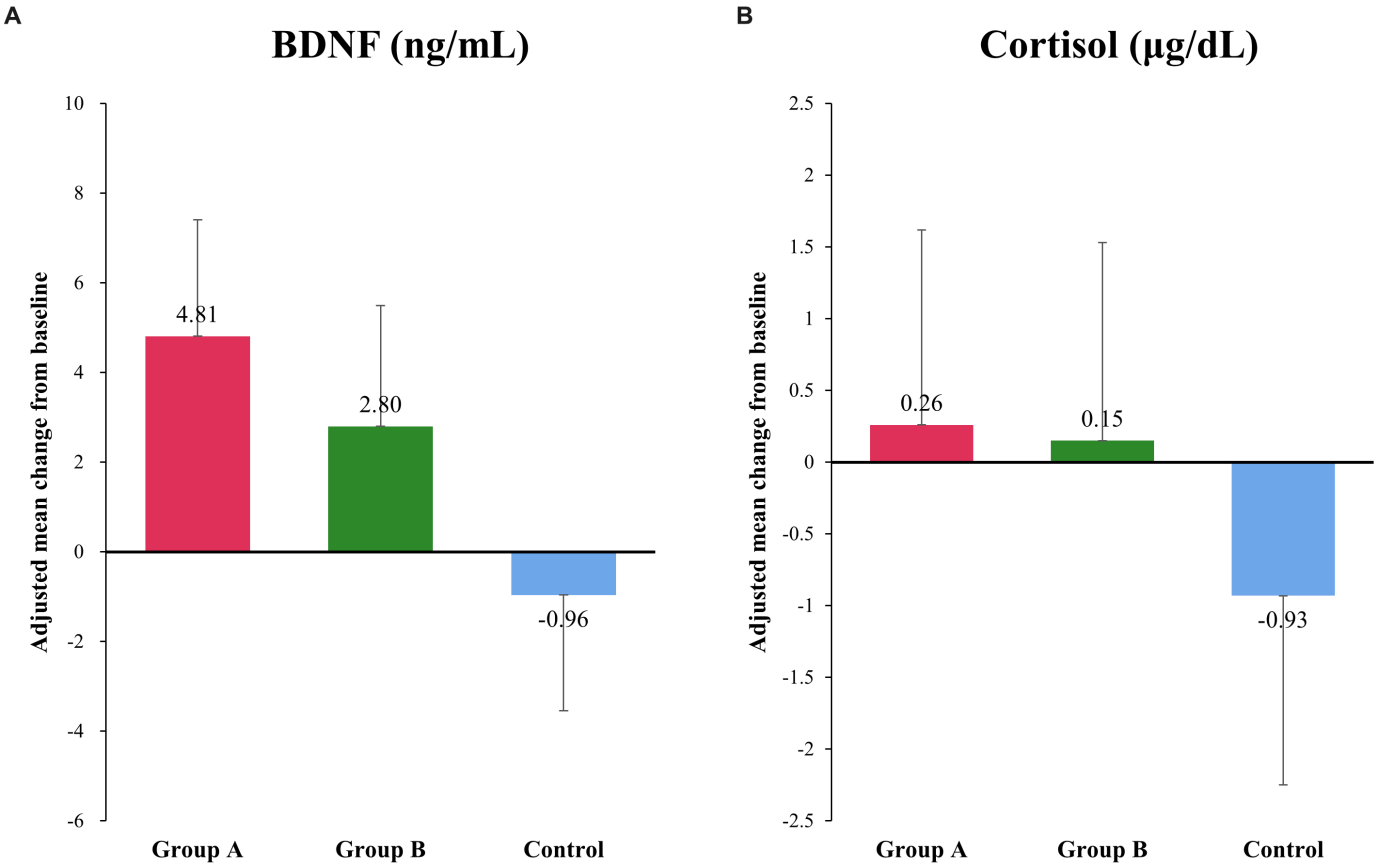
Changes in serum BDNF and plasma cortisol concentration. **(A)** There was an increase in the serum concentration of BDNF after interventions, whereas a decrease was observed in the control group, although the difference was not statistically significant. Adjusted mean (95% confidence interval) in μg/dL: group A, 4.81 (−0.44, 10.06); group B, 2.80 (−2.64, 8.23); and control group, −0.96 (−6.16, 4.24); *p* = 0.288. **(B)** There was no significant difference in the plasma cortisol level among different groups. Adjusted mean (95% confidence interval) in ng/mL: group A, 0.26 (−2.49, 3.02); group B, 0.15 (−2.67, 2.97); and control group, −0.93 (−3.6, 1.74); *p* = 0.787.

At baseline and after intervention, there were no differences in plasma cortisol and serum BDNF concentrations among the three groups (Figures 2A,B). However, after intervention, serum BDNF concentrations increased in groups A and B but decreased in the control group; these differences were not statistically significant (*p* = 0.288) (Figure 2A). There were no differences in plasma cortisol concentration among the three groups after intervention (*p* = 0.787) (Figure 2B).

**Figure 3:**
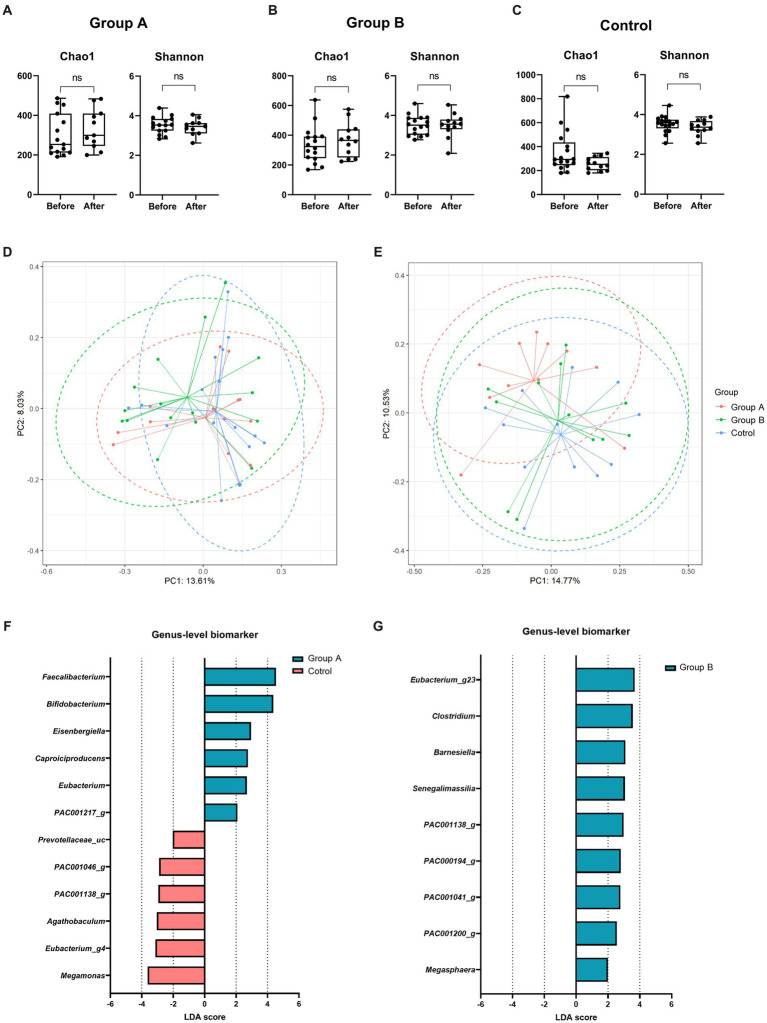
Gut microbiome analysis. There were no significant changes in alpha diversity in group A **(A)**, group B **(B)**, and the control group **(C)** before and after intervention. **(D)** The beta diversity at baseline showed no significant difference among the groups. **(E)** However, the beta diversity after intervention was significantly different between group A and the control group. **(F)** Linear discriminant analysis effect size analysis between group A and the control group showed that the genera *Faecalibacterium* and *Bifidobacterium* were more abundant in group A than in the control group after the intervention. **(G)** Group B were more enriched with Eubacterium and Clostridium than the control group, but the control group presented no characteristic genera. LDA, Linear discriminant analysis.

The gut microbiome data analysis showed that the changes in alpha diversity were not statistically different in the three groups (Figures 3A–C). PERMANOVA for beta diversity showed no differences among the three groups at baseline (*p* = 0.453) (Figure 3D). However, beta diversity after intervention was significantly different among the three groups (*p* = 0.033) and between group A and the control group (*p* = 0.013) (Figure 3E). Comparison of the linear discriminant analysis effect size between group A and the control group after intervention showed that group A was more enriched with *Faecalibacterium* and *Bifidobacterium* than was the control group (Figure 3F). After intervention, group B presented more enriched with *Eubacterium* and *Clostridium* than the control group (Figure 3G).

The gut microbiome data before and after the intervention in each experimental group were compared to that of healthy control (Supplementary material 4). After an 8-week intervention period, group A’s microbiota profile shifted toward that of the healthy control, while there were no significant changes observed in the microbiota profiles of group B and the control group (Supplementary materials 4A–E). UniFrac distances calculated between the healthy control group and each of the experimental groups also revealed that the distance was notably shorter in group A compared to the other groups (Supplementary material 4F). Moreover, *Faecalibacterium* was a genus that displayed a similar abundance between group A and the healthy control group but exhibited a significant difference in abundance when compared to the control group (Supplementary material 4G).

### 3.6. Subgroup analyses

Within MCI subgroup analyses, the results for RBANS total scale index scores and five subdomain index scores showed the similar pattern of significance as in the main analysis (Supplementary material 4). The linear mixed effects model showed a significant difference in changes in RBANS total scale index score among the three groups. The *post hoc* analysis showed that the total scale index scores improved more for group A than for group B (*p* = 0.003) and more for group A than for the control group (*p* < 0.001). The index score for the visual construction domain improved significantly in group A compared to the control group (*p* = 0.001). In the attention domain, the difference between group A and the control group was significant (*p* = 0.038). There was no significant group difference among mild dementia participants (Supplementary material 5).

Subgroup analysis for sex, showed a slightly different pattern in RBANS total scale and subdomain index scores. In the male group, changes in RBANS total scale index scores improved more for group A than for group B (*p* = 0.003) and more for group A than for the control group (*p* < 0.001), similar to the main analysis (Supplementary material 6). However, there was no significant group difference in subdomain index scores. In the female group, group A showed significant improvement in RBANS total scale index scores only compared to the control group, but not to group B (Supplementary material 7). Changes in visuoconstruction index scores of the female group were presented that group A improved significantly compared to the control group (*p* = 0.003). In addition, the attention index score showed a significant result that group A improved better than the control group (*p* = 0.023).

## 4. Discussion

To our knowledge, this is the first study to reveal the efficacy of a multidomain intervention with nutritional supplements on cognition and the gut microbiome in amyloid PET-proven early symptomatic AD patients. The RBANS total scale index scores improved significantly in patients with early symptomatic AD who received an 8-week multidomain intervention with nutritional supplements compared with patients who received nutritional supplements only or who received no intervention. Additionally, physical fitness was also significantly improved in the participants who received multidomain intervention with nutritional supplements.

Studies of multidomain interventions in patients with MCI have shown inconsistent results. The effect of multidomain interventions is difficult to decipher because of heterogeneity among the studies. Most trials that did not show the effectiveness of multidomain interventions combined only two main domains of interventions: exercise and cognitive training (Fiatarone Singh et al., 2014; Fogarty et al., 2016; Anderson-Hanley et al., 2018; Combourieu Donnezan et al., 2018; Shimada et al., 2018). In comparison, the multidomain intervention protocol of our study encompassed exercise, cognition, nutrition, and vascular risk-factor management, simultaneously providing a more comprehensive lifestyle modification to improve patient outcomes. The MAPT trial is similar to our study, as it also studied the effect of ready-to-eat nutritional supplements (omega-3 capsules) in addition to exercise and cognitive training. The subgroup analysis of patients with MCI presented positive effects; however, there was no cognitive improvement after their interventions (Andrieu et al., 2017). Thus, our study is the first trial that verifies the effectiveness of multidomain intervention with nutritional supplements on MCI and mild dementia, specifically in patients with proven Alzheimer’s pathology.

According to a previous study, among participants with mild to moderate dementia and MMSE scores ranging from 9 to 28, the RBANS total scale index score correlated well with the variety of activities of daily living (Freilich and Hyer, 2007). Therefore, significant changes in RBANS scores, particularly in group A, may signify improvements in practical functionality as perceived by patients and caregivers. However, there were no definite group differences in changes of CDR-SB or K-IADL in our study. As such, future investigations should aim to target deeper into these practical aspects to provide a more comprehensive understanding of the impact of the multidomain intervention on patients’ daily lives.

Group A showed favorable changes in attention subdomain index scores when compared to the control group. These findings align with the FINGER study, which reported enhancements in processing speed—a cognitive function closely associated with attention (Ngandu et al., 2015). This relationship is further supported by studies highlighting the effectiveness of computerized cognitive training in improving attention (Hill et al., 2017; Sherman et al., 2017), even in short-term interventions (Finn and McDonald, 2011; Hagovska and Olekszyova, 2016). Furthermore, our study underscores the positive impact of physical exercise on the attention domain (Yaguez et al., 2011). On the other hand, the best improvement of group A on visuoconstruction index score is consistent with the result of the SUPERBRAIN feasibility study that the facility-based multidomain intervention group improved on the visuoconstruction index score compared to the control group (Moon et al., 2021). However, this study showed an insignificant result in the delayed memory subdomain while the previous SUPERBRAIN study did. This difference could be explained by the different study design between both studies, and small sample size. The required sample size to evaluate the subdomains of the RBANS was found to be 13–64 participants based on the ANOVA model. Therefore, the interpretation of the subdomain index scores is limited, and a larger number of participants than in this study is required to assess significance with adequate power.

The difference between group B and the control group showed only borderline significance in our primary outcome. This is consistent with previous studies that revealed insignificant effect of providing nutritional supplements alone instead of combining with multidomain interventions (Shah et al., 2013; Soininen et al., 2017). Consequently, it is emphasized that significant effects can occur only when multidomain intervention is accompanied with nutritional supplements. However, since there was no experimental arm with the multidomain intervention alone in this study, it is not investigated whether the multidomain intervention with nutritional supplements is superior to that with the multidomain intervention alone. Therefore, further study is required to investigate the difference between the multidomain intervention with nutritional supplements and the multidomain intervention alone. Furthermore, because our studies were conducted in participants with early symptomatic AD, similar to participants in randomized controlled trials of newer disease-modifying therapies (Sims et al., 2023; van Dyck et al., 2023), future studies investigating the effects of combining multidomain interventions with disease-modifying therapy may further our understanding of AD treatment.

With cautious interpretation due to the small sample size for the RBANS subdomain index scores, this study showed borderline significance of superiority of group B over the control group for the visuoconstruction domain index score and total scale index score, while there was no difference in memory-function change between group B and the control group. In contrast, previous studies have shown that memory function improved more in patients who received nutritional supplements compared with those in control groups (Scheltens et al., 2010, 2012). This discrepancy with previous studies might be caused by the differences in the ingredients and proportions of different nutritional supplement products and the length of the intervention period.

The previous SUPERBRAIN trial revealed significant changes in biomarkers in the treatment group compared with the control group (Moon et al., 2021). However, in our study, the change in BDNF concentration among the three groups after intervention was not statistically significant. The reason might be that the number of exercise programs per week and the total number of sessions decreased from the previous study. Despite statistical insignificance, changes in BDNF concentration tended to increase in the treatment groups and decrease in the control group, which suggests a possible multidomain intervention mechanism for cognitive improvement.

Meanwhile, cortisol levels revealed no such tendency among the groups, because awakening and blood-sampling time were not strictly controlled on the examination days. Changes in the exercise protocol are also one of the reasons for the insignificant result for cortisol levels. Furthermore, some studies have suggested that physical activity, a nonpharmacological intervention, alters the dynamics of cortisol secretion rather than the cortisol concentration at a specific time point (Tortosa-Martinez et al., 2015); therefore, more sophisticated study designs and considerations are required to draw convincing conclusions from cortisol data.

The 8-week-long intervention did not change the richness and diversity scales of the gut microbiome within the groups, but it significantly changed beta diversity between group A and the control group. Moreover, microbiota of group A shifted toward that of the external healthy population. Although there is interspecies variability within a genus, *Faecalibacterium* were more abundant in group A than in the control group, and that was also the genera that showed statistically similar abundance to the external healthy population. *Bifidobacterium* was also more abundant in group A than in the control group. These are genera that are generally known to produce aminobutyric acid. The production of aminobutyric acid by some bacteria is a possible mechanism for brain-protective effects (Strandwitz, 2018). In studies in mice, exercise increased butyrate-producing bacteria (Abraham et al., 2019), and *Bifidobacterium* was depleted in mice fed high-fat diets (Nam et al., 2017; Sah et al., 2017; Sanguinetti et al., 2018). In a human study, *Bifidobacterium* was depleted in older adults (Gavini et al., 2001) and in patients with AD dementia compared with a control group (Vogt et al., 2017). These studies possibly explain why patients diagnosed with early symptomatic AD in group A had more butyrate-producing bacteria than the control group who received no intervention. Moreover, no difference in beta diversity between group B and the control group suggests that the microbiota change does not occur after simple nutrition supplementation alone. As group A showed differences in the microbiota compared with the control group, it is clear that comprehensive lifestyle modifications, including nutritional guidance, exercise, and cognitive training, are more critical for microbiota change than a simple nutritional supplement.

A low adherence rate is a common limitation of multidomain lifestyle modification studies. A recent study revealed a positive correlation between the adherence rate to the intervention and improvement in cognition (Lam et al., 2015). The improvement in outcomes might be caused by the high adherence rates in our study (>90% in all groups). There are three possible reasons for these high adherence rates. First, there was a strategy to enroll participants on the waiting list while they were motivated. All participants were educated together about dementia prevention without knowing which group they would be assigned to so that control group participants were equally motivated to participate in the program. Subsequently, the participants were informed of their assigned group, and the control group participants were enrolled on the waitlist for the same program. This maintained the motivation of the control group and gave them the expectation of receiving the multidomain intervention program at the end of the study. Second, because the program was delivered in a two-person group or as individual sessions, it was possible for trained therapists to provide a more appropriate level of training content for a participant than in large group sessions. In addition, caregivers attended the sessions and assisted the participants. Finally, a short intervention period was crucial for achieving a high adherence rate.

One limitation of our study is the relatively short intervention period compared to some other multidomain intervention trials, which have ranged from 8 weeks to 6 years (Fiatarone Singh et al., 2014; Ngandu et al., 2015; van Charante et al., 2016; Andrieu et al., 2017; McMaster et al., 2020). This shorter duration was influenced by ethical considerations, as we were mindful of the delay in treatment for participants in the control group who were waitlisted. Additionally, the ongoing COVID-19 pandemic posed challenges in terms of resource utilization, healthcare facility availability, and time constraints, which are common factors limiting the feasibility of conducting long-term studies. To address this limitation, it is essential to establish a long-term cohort study to evaluate the conversion rate from MCI to probable dementia compared with the general population.

The original SUPERBRAIN feasibility study demonstrated the effectiveness of a home-based multidomain intervention (Moon et al., 2021). However, implementing a home-based intervention in our study presented certain challenges. Notably, the cognitive stages of our study participants differed from those in the original SUPERBRAIN study, as our participants were diagnosed with MCI or mild dementia, whereas the original study included individuals with better cognitive scores. Training participants to use tablet applications for home-based interventions, as done in the original SUPERBRAIN study, was relatively straightforward. However, even in that study, it took 8 weeks to train participants to use the application independently, alongside weekly group sessions held at the facilities. Given the cognitive challenges faced by our specific population, implementing a home-based intervention within an 8-week timeframe was deemed unsuitable for achieving effective cognitive outcomes. Therefore, we chose a facility-based intervention to ensure a more structured and supervised cognitive training approach for both participants and caregivers. Consequently, while our multidomain intervention is fundamentally based on the FINGER and SUPERBRAIN protocols, we designed a new protocol tailored to our distinct target population.

Another limitation of our study is the small sample size. Several previous studies conducted in Asia have used the RBANS as the primary outcome measure, with sample sizes ranging from 48 to 98 participants per experimental arm (Cheng et al., 2012; Kita et al., 2019; Ng et al., 2018, 2021). Notably, only one study from China reported a significant intervention effect on RBANS (Cheng et al., 2012), while the majority did not. It is important to highlight that the intervention employed in the Chinese study significantly differed from ours, as it did not encompass a multidomain approach and targeted healthy elderly individuals, which is distinct from the early symptomatic AD participants in our study.

Due to the scarcity of multidomain intervention studies specifically targeting early symptomatic AD, we faced challenges in determining the required sample size *a priori*. We computed our sample size based on a study that demonstrated a significant intervention effect on RBANS, the primary outcome measure (Calapai et al., 2017). The Cohen’s f2 effect size for our primary outcome, the RBANS total scale index score, was calculated to be 0.53 (Selya et al., 2012), and its statistical power of 99.3%. Furthermore, our study adhered to the proposed criterion from a study assessing the RBANS anchor-based minimum clinically important difference (MCID) in Chinese subjects, where a difference of 8 points was considered meaningful (Phillips et al., 2015). The adjusted mean of group A met this criterion, and the proportion of the participants meeting this criterion within the group was 60.0%. This confirms the effect of the multidomain intervention with nutritional supplements. Therefore, the main results of our study are statistically reliable, despite the limitation of small sample size.

In the subgroup analysis of the MCI group, the results for the RBANS index scores showed a pattern of significance similar to that observed in the main analysis. This is because the number of MCI participants in each experimental arm is equal to or greater than eleven, which is an adequate sample size to detect an effect on the RBANS. Subgroup analysis within the mild dementia group revealed that there was no significant difference in changes in RBANS total scale index scores among the three groups. The interpretation of this result must be constrained by the critical influence of the very small sample size within the early stage of dementia groups (group A: *n* = 4, group B: *n* = 3, control: *n* = 5). In another subgroup analysis exploring the impact of sex, it was observed that Group A yielded superior results compared to group B within the male subgroup while no such distinction was evident within the female group. Nevertheless, the interpretation of this result remains constrained by the small size of the subgroups.

In this study, our multidomain intervention with nutritional supplements demonstrated notable improvements in cognition, physical performance, and the gut microbiome when compared to patients who received nutritional supplements alone or those who received no intervention. While patients who received nutritional supplements alone showed a trend toward enhanced cognition compared to those who received no intervention, the multidomain approach emerged as the most effective.

These findings offer compelling support for the use of multidomain interventions with nutritional supplements in patients with early symptomatic AD. However, to gain a more profound understanding of the intervention’s impact at specific disease stages and among different sexes, further research with larger sample sizes is warranted. Such endeavors will provide more definitive insights and contribute to advancing our knowledge of effective therapeutic strategies for this population.

## Data availability statement

The 16S rRNA gene sequencing data presented in the study are deposited in the National Center for Biotechnology Information Sequence Read Archive, accession number PRJNA1025333.

## Ethics statement

The studies involving humans were approved by Ewha Womans University, Seoul Hospital Institutional Review Board. The studies were conducted in accordance with the local legislation and institutional requirements. Written informed consent for participation in this study was provided by the participants, with the involvement of their legal guardians/next of kin.

## Author contributions

EL: Data curation, Formal analysis, Writing – review & editing, Writing – original draft. GK: Data curation, Writing – review & editing. HP: Writing – review & editing, Conceptualization, Methodology. HK: Data curation, Writing – review & editing. YKP: Data curation, Methodology, Supervision, Writing – review & editing. HL: Formal analysis, Methodology, Writing – review & editing. CH: Methodology, Supervision, Writing – review & editing. SM: Methodology, Supervision, Writing – review & editing. SC: Conceptualization, Data curation, Formal analysis, Methodology, Supervision, Writing – review & editing. JJ: Conceptualization, Data curation, Formal analysis, Methodology, Supervision, Writing – review & editing. WK: Formal analysis, Visualization, Writing - review & editing. H-SO: Formal analysis, Visualization, Writing - review & editing. H-JY: Data curation, Formal analysis, Writing – review & editing.

## Footnotes

^1^ https://www.ezbiocloud.net/contents/16smtp
