# Supplementary material for: Effects of the multidomain intervention with nutritional supplements on cognition and gut microbiome in early symptomatic Alzheimer’s disease: a randomized controlled trial
Source: Front Aging Neurosci. 2023 Nov 2;15:1266955. doi: 10.3389/fnagi.2023.1266955 (PMC10652389; doi:10.3389/fnagi.2023.1266955)
Supplement: Supplementary file 1 [file Data_Sheet_1.docx]

Supplementary Material

# Supplementary Table 1. Nutrition fact of Memory Pack Plus

| **Component** | **amount / 150 ml (150 kcal)** | **Component** | **amount / 150 ml (150 kcal)** |
| --- | --- | --- | --- |
| Carbohydrates | 20.5 g | Selenium | 60 μg |
| Dietary fiber | 3 g | Iodine | 20 μg |
| Sugars | 4g | Vitamin A | 200 μg RE |
| Fat | 6 g | Vitamin B1 | 0.26 mg |
| Saturated fat | 2 g, | Vitamin B2 | 0.3 mg |
| Trans fat | 0 g | Niacin | 15 mg NE |
| Protein | 5 g | Pantothenic  acid | 1 mg |
| Sodium | 130 mg | Vitamin B6 | 1.5 mg |
| Potassium | 125 mg | Biotin | 6 μg |
| Calcium | 100 mg | Folic acid | 400 μg |
| Phosphorus | 90 mg | Vitamin B12 | 3 μg |
| Magnesium | 25 mg | Vitamin C | 40 mg |
| Iron | 2.4 mg | Vitamin D | 1 μg |
| Zinc | 1.5 mg | Vitamin E | 20 mg α-TE |
| Other components*: Eicosapentaenoic acid (EPA) and docosahexaenoic acid (DHA) containing fat, medium-chain triglycerides (MCT) oil, phosphatidyl serine, disodium 5'-uridylate | | | |

*Information about the amount per serve is not available due to the policy of the Daesang Life Science Corporation that provided the products.

RE, retinol equivalent; NE, niacin equivalent; α-TE, alpha tocopherol equivalent.

# Protocol of multidomain intervention for the present study

## Vascular risk factors control

At the beginning of the intervention, a face-to-face education session was conducted by a study nurse. All participants were educated about vascular risk factors and lifestyle guidelines for dementia prevention.

Every 4 weeks, the study nurse assessed alcohol consumption and smoking status and measured anthropometric data, including blood pressure, body weight, and abdominal circumference. These measurements were reported individually to each participant and were recorded in the SUPERBRAIN participant’s notebook at each measurement visit.

## Cognitive training

Cognitive training was delivered by trained health professionals (occupational therapists and study nurses) in individual sessions twice per week for 50 minutes per session during the 8-week intervention period. Structured diary writing was assigned as homework.

The session consisted of three main parts: welcome and homework review (10 minutes), main training (35 minutes), and wrap-up (5 minutes). During the main training part, one memory task (20 minutes), two frontal executive function/attention tasks (10 minutes), and one visuospatial or calculation task (5 minutes) were provided. In this study, all participants received cognitive training using a tablet-based application.

The tablet-based cognitive training application contained 48 memory tasks, 12 frontal executive function/attention tasks, 4 visuospatial tasks, and 4 arithmetic tasks. Except for the memory tasks, each task category had difficulty levels ranging from level 1 (the easiest) to level 10 (the most difficult). The appropriate level of the tasks was flexibly determined by trained health professionals who assessed each participant’s performance on the tasks at each session.

### Memory tasks

he memory training consisted of three steps. First, participants were presented with information about a topic. Second, participants were encouraged to remember the topic using memory strategies and process-based training. Spaced retrieval, visual imagery, story making, association, chunking, categorization, sequencing, acronyms, keyword method, rehearsal, and rhyming were trained as memory strategies. Finally, quizzes were administered to check whether the participants were successful in memorization.

The topics of the memory tasks were as follows: Korean folk tales and myths, history and characteristics of Jeju Island and Gangwon Province, health knowledge (dental hygiene, stroke, dementia-prevention strategies, Alzheimer’s disease, sleep, and air pollution), local festivals, foreign greetings, food (brain foods, seasonal foods, history of kimchi, and traditional Korean liquors by region), and national flags.

### Frontal executive function and attention tasks

Each task was preceded by a tutorial. The main tasks were performed on the basis of process-based training. The 12 different contents of this category of tasks are as follows: placing footprints forward or backward, placing numbers in ascending or descending order, placing all pictures with the presented features, tapping blocks in the presented order, catching fish with suggested features among various fish, picking up fruits as fast as possible, the *n*-back task, sudoku, performing the directed tasks by remembering where tomato sprouts disappeared from, and finding matching cards from multiple face-down cards.

### Visuospatial function

Each task was preceded by a tutorial. The main tasks were performed on the basis of process-based training. The content of this category was as follows: following arrows to find a treasure or an acorn, visualizing a picture as two pictures combined, and finding the same figures oriented in different directions.

### Calculation

Each task was preceded by a tutorial. The main tasks were performed on the basis of process-based training. The four computation tasks were as follows: calculating arrival time, breaking honeycomb by calculations, summing after rotating numbers, and calculating the total cost of shopping items.

### Diary

The diary was assigned as homework. It had a structured format with seven specific questions. The questions included recording the date and weather for the day, expressing personal feelings, documenting meals consumed, noting people met and places visited (including method and purpose of travel), tracking expenses with specific details, reflecting on current news and personal thoughts, and outlining plans for the following day. The purpose of this activity was to improve cognitive skills such as orientation, mindfulness, episodic memory, healthy eating habits, mathematical skills, and prospective memory.

## Physical exercise

The exercise session was conducted as a two-person group session. The session consisted of aerobic exercise, balance and flexibility exercises, muscle strengthening activities involving major muscle groups, and finger and toe exercises. Trained exercise professionals conducted the sessions in a gymnasium. Portable equipment, such as rubber bands, nine numbered floor mats, and chairs, were used for the sessions.

The structure of the exercise session was based on the protocol of the previous feasibility study [14]. However, to improve adherence to the overall intervention, the number of exercise sessions was reduced to twice per week, and they were scheduled on the same day as the cognitive training sessions. In addition, because the duration of this trial was only 8 weeks, the exercise program was maintained at level 1 of the previous feasibility study.

The level 1 program aimed for 40% to 50% of maximum heart rate during the session. It consisted of resistance exercise (20–25 minutes), aerobic exercise (20–25 minutes), balance exercise (5 minutes), finger and toe movement (5 minutes), and stretching (5 minutes). The number of muscle groups used in the resistance exercises was 10, and the number of resistance exercises was one or two.

Exercise programs were individualized for each participant based on their baseline physical performance and condition. There were four options, including a strength-intensive program and an aerobic-exercise program specifically designed for individuals with good physical fitness, and a strength-intensive program and an aerobic-exercise program specifically designed for individuals with poor physical fitness. Poor physical fitness was defined as a score below the 30th percentile of norms for the same age and sex on any physical performance test.

## Nutrition

Individual nutritional guidance sessions by study nutritionists were conducted once every 3 weeks. Each session lasted 30 minutes and included a review of the participant’s daily diet, education on individualized diets to manage vascular risk factors, practical activities to facilitate dietary changes, and advice on how to prepare meals using recommended ingredients.

The guidelines were based on the MIND diet [50]. Participants were advised to eat at least three servings of whole grains, at least one dark-green salad, one additional vegetable, and one ounce of nuts per day. It was recommended that they include beans or legumes in their meals every other day, eat berries and poultry at least twice per week, consume fish at least once per week, and drink a glass of wine daily. Olive oil was recommended as a healthier alternative to butter or margarine. Cheese, fried foods, and fast foods were limited to no more than once per week. Finally, pastries and sweet foods were limited to no more than five times per week. In addition, participants completed the MIND diet checklist every 2 weeks to stay motivated.

## Motivation

At the beginning of the intervention, there was an in-person session with a study physician to encourage commitment to the entire intervention. The study coordinator sent weekly text messages to encourage study participation and daily lifestyle adjustments.

# Supplementary Table 2. Information about acetylcholine esterase inhibitor intake

|  | **Group A**  **(n=15)** | **Group B**  **(n=15)** | **Control**  **(n=16)** | ***p*** |
| --- | --- | --- | --- | --- |
| **AChEI use** | 13 (86.7) | 14 (93.3) | 16 (100) | 0.323 |
| **Donepezil** |  |  |  |  |
| 2.5mg | 1 (6.67) | 0 (0.00) | 4 (25.00) |  |
| 5mg | 5 (33.33) | 7 (46.67) | 3 (18.75) |  |
| 7.5mg | 1 (6.67) | 0 (0.00) | 3 (18.75) |  |
| 10mg | 5 (33.33) | 6 (40.00) | 5 (31.25) |  |
| 12.5mg | 1 (6.67) | 1 (6.67) | 1 (6.25) |  |
| **Mean dose, mg*** | 5.17 (4.17) | 7.17 (3.39) | 6.25 (3.65) |  |

Values are expressed as number (percent), except where noted otherwise

*Values are expressed as mean (standard deviation)

AChEI, acetylcholine esterase inhibitor

# Supplementary Figure 1. Gut microbiome analysis comparing experimental groups to an external healthy population cohort

**(A)**The microbiota of group A shifted toward that of the healthy control after 8 weeks of intervention, **(B)** while the microbiota of group B and **(C)** the control group did not significantly shift toward that of the healthy control. **(D)** Changes in the distance to the healthy control were significantly reduced in group A, while there were no significant changes in group B. In the control group, the distance to the healthy control actually became longer after the intervention period than before. **(E)** When simultaneously plot the result of each group after intervention and healthy control, the shift of group A toward the healthy control is clearly demonstrated. **(F)** UniFrac distances between the healthy control and each group also showed that the distance is significantly shorter in group A than other groups. **(G)** *Faecalibacterium* was the genera that showed similar abundance between group A and the healthy control group, while significantly different in abundance between these two groups and the control group.

*, p<0.05; **, p<0.01; ***, p<0.001; ns, not significant

# Supplementary table 3. Subgroup analysis - MCI group

|  | **Baseline** | | | | **Changes from baseline to study end** | | | | | |
| --- | --- | --- | --- | --- | --- | --- | --- | --- | --- | --- |
|  | **Group A**  **(n=11)** | **Group B**  **(n=12)** | **Control**  **(n=11)** | ***p*** | **Group A**  **(n=11)** | **Group B**  **(n=12)** | **Control**  **(n=11)** |  | | |
|  |  |  |  |  |  |  |  | **A vs B** | **A vs C** | **B vs C** |
| **Total scale index score** | 80.82 (16.88) | 78.17 (9.45) | 80.09 (17.05) | 0.905 | 10.82  (6.33, 15.31) | 0.92  (-3.38, 5.21) | -4.55  (-9.03, -0.06) | 0.003* | <0.001* | 0.083 |
| **Immediate memory** | 83.27 (12.93) | 76.25 (10.12) | 84.36 (15.62) | 0.278 | 7.64  (0.81, 14.47) | 3.25  (-3.29, 9.79) | -1.27  (-8.1, 5.56) | 0.351 | 0.069 | 0.337 |
| **Delayed memory** | 71.09 (21.32) | 62.92 (19.10) | 68.91 (18.03) | 0.585 | 8.36  (0.49, 16.24) | 3.17  (-4.37, 10.71) | -2  (-9.88, 5.88) | 0.339 | 0.067 | 0.341 |
| **Visuo-construction** | 98.64 (15.72) | 101.75 (6.58) | 94.00 (16.92) | 0.407 | 3.55  (-1.77, 8.86) | -3.5  (-8.59, 1.59) | -9.82  (-15.13, -4.5) | 0.06 | 0.001 | 0.09 |
| **Language** | 88.18 (13.36) | 89.17 (13.79) | 94.00 (10.65) | 0.521 | 8.64  (1.87, 15.4) | 6.33  (-0.14, 12.81) | 0.36  (-6.4, 7.13) | 0.619 | 0.088 | 0.203 |
| **Attention** | 92.36 (13.89) | 94.50 (7.33) | 90.27 (14.71) | 0.715 | 6.73  (0.97, 12.48) | -1.08  (-6.59, 4.43) | -1.91  (-7.67, 3.85) | 0.054 | 0.038 | 0.834 |

Values of baseline index scores are expressed as mean (standard deviation). Values of the changes in index scores are expressed as the adjusted mean (95% CI).

* p<0.05

† 0.05< p < 0.1, borderline significance

AvsB, group A versus group B; AvsC, group A versus control group; BvsC, group B versus control group; CI: confidence interval; RBANS, Repeatable Battery for the Assessment of Neuropsychological Status

# Supplementary table 4. Subgroup analysis – Mild dementia group

|  | **Baseline** | | | | **Changes from baseline to study end** | | | | | |
| --- | --- | --- | --- | --- | --- | --- | --- | --- | --- | --- |
|  | **Group A**  **(n=4)** | **Group B**  **(n=3)** | **Control**  **(n=5)** | ***p*** | **Group A**  **(n=4)** | **Group B**  **(n=3)** | **Control**  **(n=5)** |  | | |
|  |  |  |  |  |  |  |  | **A vs B** | **A vs C** | **B vs C** |
| **Total scale index score** | 72.50 (17.75) | 60.67 (8.62) | 78.20 (14.70) | 0.312 | 4.00  (-4.58, 12.58) | 5.33  (-4.57, 15.24) | -4.60  (-12.27, 3.07) | 0.823 | 0.125 | 0.107 |
| **Immediate memory** | 75.00 (11.92) | 67.67 (14.57) | 76.20 (10.52) | 0.616 | -1.50  (-14.41, 11.41) | 8.67  (-6.24, 23.57) | 1.00  (-10.54, 12.54) | 0.273 | 0.751 | 0.382 |
| **Delayed memory** | 61.00 (15.85) | 56.67 (10.26) | 55.00 (12.57) | 0.798 | 5.25  (-3.97, 14.47) | 6.00  (-4.65, 16.65) | 4.60  (-3.65, 12.85) | 0.907 | 0.908 | 0.819 |
| **Visuo-construction** | 100.00 (14.07) | 83.00 (21.70) | 106.00 (9.25) | 0.144 | 4.50  (-11.29, 20.29) | -3.00  (-21.24, 15.24) | -15.00  (-29.13, -0.87) | 0.5 | 0.067 | 0.27 |
| **Language** | 79.50 (14.84) | 80.00 (3.46) | 93.40 (7.16) | 0.116 | -0.50  (-9.36, 8.36) | 5.33  (-4.89, 15.56) | -6.00  (-13.92, 1.92) | 0.355 | 0.322 | 0.079 |
| **Attention** | 91.75 (11.06) | 81.33 (15.95) | 94.00 (17.59) | 0.533 | 4.25  (-4.08, 12.58) | 0.33  (-9.29, 9.96) | 0.60  (-6.85, 8.05) | 0.504 | 0.479 | 0.962 |

Values of baseline index scores are expressed as mean (standard deviation). Values of the changes in index scores are expressed as the adjusted mean (95% CI).

* p<0.05

† 0.05< p < 0.1, borderline significance

AvsB, group A versus group B; AvsC, group A versus control group; BvsC, group B versus control group; CI: confidence interval; RBANS, Repeatable Battery for the Assessment of Neuropsychological Status

# Supplementary table 5. Subgroup analysis - Male group

|  | **Baseline** | | | | **Changes from baseline to study end** | | | | | |
| --- | --- | --- | --- | --- | --- | --- | --- | --- | --- | --- |
|  | **Group A**  **(n=6)** | **Group B**  **(n=10)** | **Control**  **(n=4)** | ***p*** | **Group A**  **(n=6)** | **Group B**  **(n=10)** | **Control**  **(n=4)** |  | | |
|  |  |  |  |  |  |  |  | **A vs B** | **A vs C** | **B vs C** |
| **Total scale index score** | 75.33 (16.21) | 77.10 (12.41) | 80.50 (9.11) | 0.832 | 11.50  (6.83, 16.17) | 1.50  (-2.11, 5.11) | 2.50  (-3.22, 8.22) | 0.002 | 0.020 | 0.759 |
| **Immediate memory** | 79.33 (15.51) | 78.00 (10.21) | 75.75 (7.63) | 0.894 | 3.33  (-7.37, 14.04) | 2.80  (-5.49, 11.09) | 5.00  (-8.11, 18.11) | 0.935 | 0.838 | 0.768 |
| **Delayed memory** | 62.83 (24.23) | 66.50 (19.14) | 58.25 (12.82) | 0.778 | 14.00  (3.54, 24.46) | 3.80  (-4.3, 11.9) | 1.25  (-11.56, 14.06) | 0.122 | 0.122 | 0.727 |
| **Visuo-construction** | 96.50 (7.68) | 96.40 (14.89) | 106.00 (10.10) | 0.403 | 7.50  (-0.24, 15.24) | -2.10  (-8.1, 3.9) | -4.50  (-13.98, 4.98) | 0.054 | 0.054 | 0.657 |
| **Language** | 85.17 (9.99) | 88.40 (10.75) | 95.50 (5.20) | 0.282 | 6.67  (-2.83, 16.17) | 7.40  (0.04, 14.76) | 3.00  (-8.63, 14.63) | 0.899 | 0.613 | 0.509 |
| **Attention** | 92.17 (10.15) | 92.00  (6.83) | 96.00 (15.98) | 0.783 | 5.00  (-2.21, 12.21) | -1.30  (-6.88, 4.28) | 4.25  (-4.58, 13.08) | 0.163 | 0.891 | 0.278 |

Values of baseline index scores are expressed as mean (standard deviation). Values of the changes in index scores are expressed as the adjusted mean (95% CI).

* p<0.05

† 0.05< p < 0.1, borderline significance

AvsB, group A versus group B; AvsC, group A versus control group; BvsC, group B versus control group; CI: confidence interval; RBANS, Repeatable Battery for the Assessment of Neuropsychological Status

# Supplementary table 6. Subgroup analysis - Female group

|  | **Baseline** | | | | **Changes from baseline to study end** | | | | | |
| --- | --- | --- | --- | --- | --- | --- | --- | --- | --- | --- |
|  | **Group A**  **(n=9)** | **Group B**  **(n=5)** | **Control**  **(n=12)** | ***p*** | **Group A**  **(n=6)** | **Group B**  **(n=10)** | **Control**  **(n=4)** |  | | |
|  |  |  |  |  |  |  |  | **A vs B** | **A vs C** | **B vs C** |
| **Total scale index score** | 80.78 (17.94) | 69.80 (8.67) | 79.17 (17.91) | 0.490 | 7.33  (1.72, 12.94) | 2.40  (-5.13, 9.93) | -6.92  (-11.77, -2.06) | 0.288 | 0.001 | 0.042 |
| **Immediate memory** | 82.22 (11.54) | 67.60 (10.36) | 83.83 (15.79) | 0.090 | 6.44  (-0.65, 13.54) | 7.40  (-2.11, 16.91) | -2.42  (-8.56, 3.72) | 0.869 | 0.063 | 0.086 |
| **Delayed memory** | 72.11 (17.16) | 52.00  (8.94) | 66.67 (18.69) | 0.120 | 3.22  (-4.58, 11.03) | 3.60  (-6.87, 14.07) | -0.33  (-7.09, 6.43) | 0.953 | 0.483 | 0.52 |
| **Visuo-construction** | 100.67 (18.42) | 101.20 (6.91) | 95.00 (16.60) | 0.655 | 1.33  (-5.64, 8.3) | -6.00  (-15.35, 3.35) | -13.75  (-19.79, -7.71) | 0.206 | 0.003 | 0.163 |
| **Language** | 86.33 (16.44) | 85.20 (17.63) | 93.25 (10.64) | 0.434 | 5.89  (-1.24, 13.01) | 3.60  (-5.96, 13.16) | -3.17  (-9.34, 3) | 0.695 | 0.059 | 0.231 |
| **Attention** | 92.22 (14.93) | 91.60 (16.55) | 89.92 (15.29) | 0.940 | 6.78  (0.53, 13.02) | 0.20  (-8.18, 8.58) | -2.92  (-8.32, 2.49) | 0.206 | 0.023 | 0.524 |

Values of baseline index scores are expressed as mean (standard deviation). Values of the changes in index scores are expressed as the adjusted mean (95% CI).

* p<0.05

† 0.05< p < 0.1, borderline significance

AvsB, group A versus group B; AvsC, group A versus control group; BvsC, group B versus control group; CI: confidence interval; RBANS, Repeatable Battery for the Assessment of Neuropsychological Status
