# Supplementary figures and images for: Effects of the multidomain intervention with nutritional supplements on cognition and gut microbiome in early symptomatic Alzheimer’s disease: a randomized controlled trial
Source: Front Aging Neurosci. 2023 Nov 2;15:1266955. doi: 10.3389/fnagi.2023.1266955 (PMC10652389; doi:10.3389/fnagi.2023.1266955)

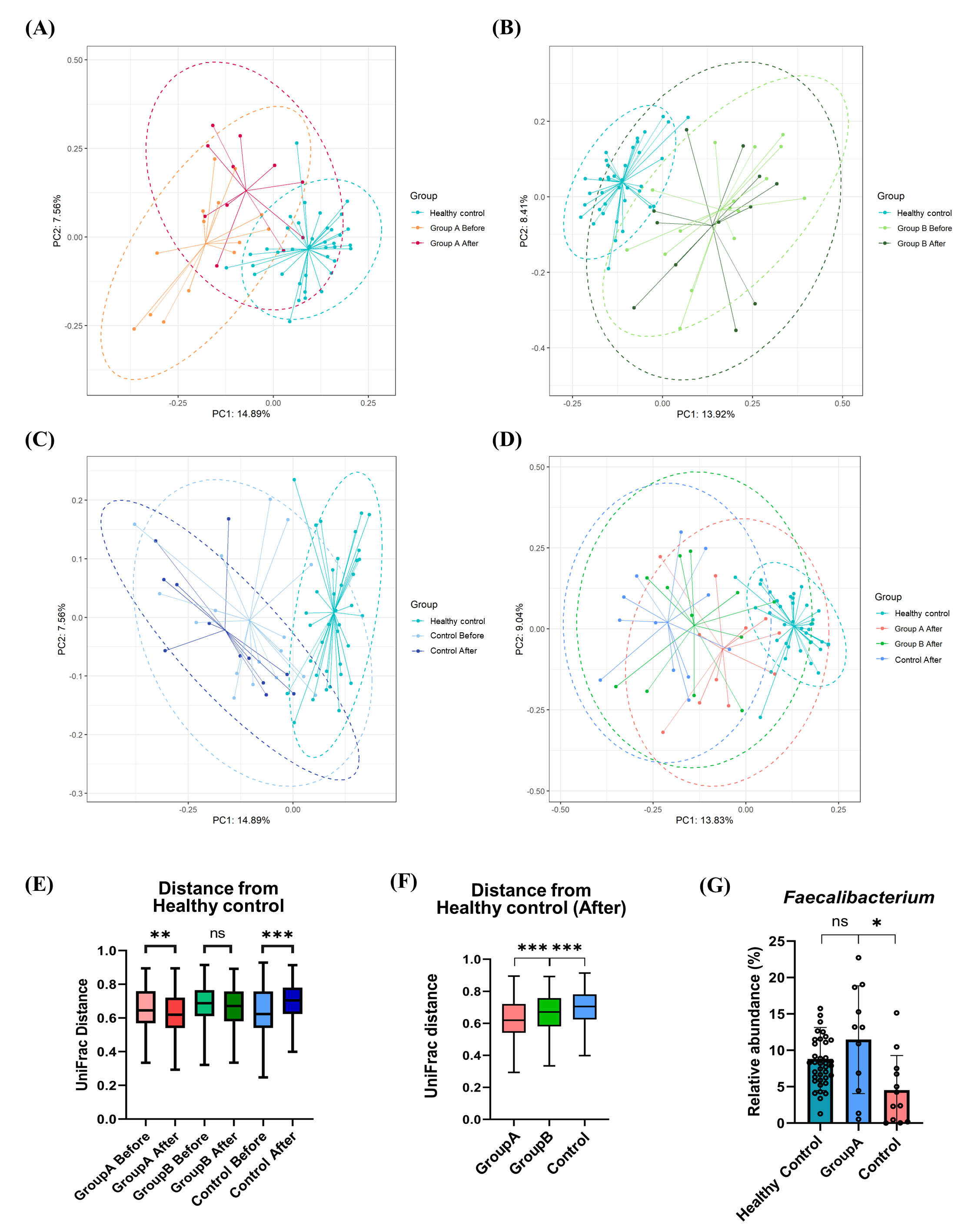

Supplement: Supplementary file 2 [file Image_1.TIF]
